# Shoulder and thorax kinematics contribute to increased power output of competitive handcyclists

**DOI:** 10.1111/sms.13402

**Published:** 2019-03-18

**Authors:** Benjamin Stone, Barry S. Mason, Martin B. Warner, Victoria L. Goosey‐Tolfrey

**Affiliations:** ^1^ Peter Harrison Centre of Disability Sport, School of Sport, Exercise and Health Sciences Loughborough University Loughborough UK; ^2^ School of Health Sciences University of Southampton Southampton UK; ^3^ Arthritis Research UK Centre for Sport Exercise and Osteoarthritis Nottingham UK

**Keywords:** disability sport, kinematics, recumbent handcycling, statistical parametric mapping

## Abstract

Current knowledge of recumbent handbike configuration and handcycling technique is limited. The purpose of this study was to evaluate and compare the upper limb kinematics and handbike configurations of recreational and competitive recumbent handcyclists, during sport‐specific intensities. Thirteen handcyclists were divided into two significantly different groups based on peak aerobic power output (PO_peak_) and race experience; competitive (n = 7; 5 H3 and 2 H4 classes; PO_peak_: 247 ± 20 W) and recreational (n = 6; 4 H3 and 2 H4 classes; PO_peak_: 198 ± 21 W). Participants performed bouts of exercise at training (50% PO_peak_), competition (70% PO_peak_), and sprint intensity while three‐dimensional kinematic data (thorax, scapula, shoulder, elbow, and wrist) were collected. Statistical parametric mapping was used to compare the kinematics of competitive and recreational handcyclists. Handbike configurations were determined from additional markers on the handbike. Competitive handcyclists flexed their thorax (~5°, *P* < 0.05), extended their shoulder (~10°, *P* < 0.01), and posteriorly tilted their scapular (~15°, *P* < 0.05) more than recreational handcyclists. Differences in scapular motion occurred only at training intensity while differences in shoulder extension and thorax flexion occurred both at training and competition intensities. No differences were observed during sprinting. No significant differences in handbike configuration were identified. This study is the first to compare the upper limb kinematics of competitive recreational handcyclists at sport‐specific intensities. Competitive handcyclists employed significantly different propulsion strategies at training and competition intensities. Since no differences in handbike configuration were identified, these kinematic differences could be due to technical training adaptations potentially optimizing muscle recruitment or force generation of the arm.

## 1 INTRODUCTION

Handcycling is an activity that has increased in popularity at both a recreational and sporting level.^1^ At the 2016 Paralympic Games, handcycling contributed 39% of the events in the road cycling programme^2^ and was incorporated within the wheelchair class of paratriathlon. In the sport of handcycling, athletes are classified into one of five categories (H1‐H5) according to the nature of their impairment, with H1 being most impaired.^3^ Athletes in classes H1‐H4 use arm‐powered recumbent handbikes and the H5 class, because of their greater functional capacity, use arm‐trunk‐powered kneeling handbikes.^4^, ^5^ Handcycling is typically classed as an endurance sport,^6^ where athletes compete in time trial and road race disciplines, lasting 20‐140 minutes, but due to the race terrains and tactics speeds average 30‐45 km/h with a top speed of ~55 km/h being noted.^6^, ^7^

While the physiological performance determinants of recumbent handcycling have been investigated extensively,^6^, ^8^, ^9^ very little is known about handcycling biomechanics or handbike configuration, which is critical for the development of the sport from both a performance and injury perspective. It has been established in able‐bodied cycling that components of the bike and bike‐rider interface impact technique and potentially sports performance.^10^ Of the handcycling biomechanical literature, studies have established that factors such as exercise intensity^11^, ^12^ and handbike configuration^13^, ^14^ affect handcycling technique. However, these studies predominantly used able‐bodied or inexperienced handcyclists, exercising at ambulatory intensities using recreational (upright) handbikes, which limits the transferability to the sport of recumbent handcycling.^5^ Recumbent handcycling is a very specific upper body exercise modality performed by a very specific group of athletes. The use of able‐bodied or less experienced participants can bias the findings, for example, in the context of wheelchair propulsion “skilled users” push with higher efficiencies^15^ and following training or familiarization display improved techniques.^16^, ^17^ Therefore, factors such as the participant's skill level and other characteristics of the experimental study such as the exercise intensity and handbike configuration need to be carefully considered.

To study recumbent handcycling technique under realistic conditions, three‐dimensional (3D) kinematic analysis is preferred. Presently, only two‐dimensional approaches of the elbow and wrist kinematics have been employed,^5^ not taking into account the scapular, shoulder, or thorax kinematics. Simplistic summary metrics, such as range of motion (RoM), joint minimum, and joint maximum, have been used to quantify performance or changes in technique in handcycling.^5^, ^11^, ^18^, ^19^ The use of these outputs results in regional focus bias, as only two or three data points are considered from the whole kinematic trajectory.^20^ Additionally, the temporal characteristics of the minimum and maximum joint angles are often not considered. Identifying where in the cycle technical differences exist will further our understanding of handcycling technique and potentially explain the relationships between handcycling technique, handbike configuration, injury risk, and sports performance.

The current study compared 3D upper limb kinematics (thorax, scapula, upper arm, forearm, hand) of recumbent handcyclists differing in performance level (competitive: vs recreational:) under realistic sports conditions. Statistical parametric mapping (SPM) was employed to provide an insight into the differences in technique between groups. Recumbent handbike configuration, the configuration of the handbike relative to users anthropometry and the functional capacity of the participants are likely to influence handcycling technique, so these factors were compared between groups. Identifying recumbent handbike configuration and technical differences between performance levels could help coaches and clinicians identify key characteristics of configuration or technique, potentially leading to improved performance or reduced injury risk.

## 2 METHODS

### 2.1 Participants

Thirteen trained male recumbent handcyclists (mean ± standard deviation (SD); age: 37.6 ± 8.6 years; body mass: 70.5 ± 9.6 kg) participated in the study. Participants were divided into two distinct performance groups (competitive and recreational) based on their racing level and peak aerobic power output (PO_peak_), shown as the best predictor of handcycling time trial performance in our laboratory and in a previous study.^21^ Group mean PO_peak_ was calculated (223 W) and participants with a low PO_peak_ (<223 W) were grouped as recreational and participants with a high PO_peak_ (>223 W) were grouped as competitive (Table 1). This resulted in seven handcyclists, with international racing experience, in the competitive group (classification: 5 H3 and 2 H4; training load: 13 ± 2 h/wk; handbike: 4 Top End, 1 Carbonbike, 1 Schmicking, and 1 Wolturnus) and six handcyclists in the recreational group (classification: 4 H3 and 2 H4; training load: 10 ± 2 h/wk; handbike: 3 Top End, 2 Carbonbike, and 1 Schmicking). Ethical approval for the study was obtained from the university's local ethics committee. Before participation, all athletes provided their written, informed consent.

**Table 1:** Physiological characteristics of competitive and recreational handcyclists, determined in submaximal and maximal incremental exercise test (values are Mean ± SD)

Parameter	Competitive	Recreational	ES
Sub‐maximal test			
Aerobic threshold (W)	98 ± 19*	56 ± 17	4.21
Anaerobic threshold (W)	137 ± 15*	91 ± 21	4.20
Maximal Test			
V̇O_2peak_ (L/min)	3.17 ± 0.34*	2.57 ± 0.19	0.52
V̇O_2peak_ (mL/kg/min)	45.04 ± 5.84**	37.26 ± 6.47	2.48
Peak power (W)	247 ± 20*	198 ± 21	4.49
Peak HR (bpm)	188 ± 7	183 ± 9	2.81

ES, effect size.

* *P* < 0.005.

** *P* < 0.05.

### 2.2 Experimental protocol

Participants attended the laboratory for two exercise bouts over the course of one day. All exercise bouts were completed at a self‐selected cadence and in the participants' recumbent handbikes which were attached to an ergometer (Cyclus 2, Richter, Germany). Firstly, the participants completed a sub‐maximal incremental exercise test. The protocol started at 20 W with resistance increasing by 20 W every four minutes. The sub‐maximal exercise test was terminated when the participant's blood lactate concentration reached 4 mmol/L. Small capillary blood samples, obtained from the earlobe, were collected in the last 60 seconds of each stage. Blood samples were analyzed and disposed of immediately (Biosen C Line Monitor, EKF Diagnostics, Barleben, Germany). Following a 15 min rest period, the participants performed a maximal test to exhaustion. Participants commenced handcycling at the power output equivalent to their aerobic threshold, obtained during the sub‐maximal exercise protocol using a log‐log transformation method,^22^ for two minutes at a self‐selected cadence. Resistance increased by 5 W every 15 seconds until the participant reached volitional exhaustion (failure to maintain cadence ≥50 rpm).^23^ Breath‐by‐breath gas analysis (Cortex Metalyzer 3B, Cortex, Leipzig, Germany) and heart rate (HR) (Polar RS400, Kempele, Finland) were collected throughout the submaximal and maximal protocols. After 2 hours of recovery, participants completed two 5 minute exercise bouts, in a randomized order, at a power output equivalent to training (50% PO_peak_) and competition intensity (70% PO_peak_),^6^, ^7^ with 5 minutes rest between trials. Following the completion of the second bout, participants rested for a further 20 minutes, before completing a 20 seconds sprint test.^4^ The sprint started from a rolling start of 70 rpm, using a resistance equivalent to 5% body weight.^24^ Cadence and power output (Cyclus 2) were collected throughout the training, competition, and sprint exercise bouts. The Cyclus 2 has a maximal error of 2% when measuring power and ±1 rpm with cadence, for further information the reader is directed to the manufacturer website.

### 2.3 Kinematics

A motion capture system (Vicon Motion Systems, Oxford, UK) consisting of 10 T40S cameras, sampling at 200 Hz, was used to capture upper limb kinematics during the training, competition, and sprint intensities. Retroreflective markers were attached to the thorax (C7, T8, incisura jugularis and xiphoid process), the right forearm (radial styloid and ulnar styloid) and bilaterally to the left and right hand (2nd and 5th carpometacarpal (CMC) and metacarpophalangeal joints) in accordance with the International Society of Biomechanics (ISB) recommendations.^25^ Clusters were attached to the acromions,^26^, ^27^ the right upper arm (12 markers), and forearm (2 markers) to track the scapula, humerus, and forearm, respectively. The acromion marker cluster technique for estimating scapular kinematics has previously been shown to be valid and reliable in static and dynamic conditions in the sagittal and scapular plane (0°‐90°).^26^, ^28^ Additional thorax markers were attached, a three‐marker‐cluster attached to the sternum and bilateral markers on the 10th rib, as C7 and T8 markers were removed during the handcycling trials due to marker occlusion caused by the participant's recumbent position.^11^ Markers were also attached bilaterally to the crank arms of the participant's handbike.

The participant sat in the anatomical position while thirteen, three‐second static anatomical landmark trials were performed, to determine the anatomical landmarks of the scapula and humerus with respect to the marker clusters. The tip of a calibration wand was placed onto the anatomical landmarks of the sternoclavicular joint (SC), acromioclavicular joint (AC), acromion angle (AA), the root of the medial spine (TS), inferior angle (AI) and the right humeral lateral epicondyle (EL) and medial epicondyle (EM).^29^ Participants then performed a 10‐second shoulder circumduction trial to determine the glenohumeral joint center functionally.^30^

### 2.4 Kinematic analysis

The Optimal Common Shape Technique^31^ was utilized for the markers on the thorax, upper arms, forearms, and hands to account for soft tissue artefact. The process involved determining the common shape of the markers for each given segment during the shoulder circumduction trial using a Generalised Procrustes Analysis. The common shape was then mapped onto the respective segments through the utilization of an Ordinary Procrustes Analysis for all other trials. The right glenohumeral joint center was determined using the Symmetrical Centre of Rotation Estimation^32^ technique during the circumduction calibration trial. The anatomical landmarks of thorax (SC, C7 & T8), acromion cluster (AC, AA, TS, and AI), and upper arm (EM and EL) were reconstructed in the global coordinate system during the dynamic trials based on their known location with respect to the marker clusters determined during the anatomical landmark trials.^33^ The global coordinate system was defined such that the Y‐axis pointed anteriorly, the X‐axis aligned with the rotation axis of the crank, and the Z‐axis pointed vertically following the right‐hand rule. Anatomical local coordinate systems and rotation sequences for the thorax, clavicle, scapula, humerus, forearm, and hand were then constructed, in accordance with ISB recommendations.^25^

In line with previous research, upper limb kinematics were analyzed over ten consecutive cycles.^34^ A cycle was defined as one rotation of the crank, starting with the cranks in a vertical position pointing up. A crank local coordinate system, that aligned with the global Z‐axis, was created using the crank arm marker and the center of rotation of the crank axis, which was calculated using a sphere fitting method.^35^ Crank angle was then determined using Euler angles (ZXY sequence). Upper limb kinematics were normalized to cycle duration (0%‐100%) and then averaged across ten cycles.^36^ These average cycles were then inputted used in the SPM analysis.

### 2.5 Anthropometrics and handbike configuration

Anthropometrics, arm length (AA to the 5th CMC), and shoulder breadth (left AA to right AA), were calculated in a static trial. Handbike configuration and handbike‐user interface were determined during the dynamic trials. Shoulder and crank height were calculated from the height of AA and crank center. Handgrip width was calculated as the distance between the center of the four markers on the left and right hand. Crank fore‐aft position was calculated as the distance between AA and 5th CMC when the cranks are parallel to the floor and pointing away from the athlete's chest. The configuration of the handbike‐user interface was then calculated using crank height, crank width, and crank fore‐aft position about shoulder height, shoulder breadth, and arm length.

### 2.6 Statistical analysis

To assess differences in economy, power output, cadence, anthropometry, and handbike configuration between competitive and recreational handcyclists, independent *t* tests were employed. Corrected effect sizes (ES), for independent samples with unequal sample sizes, were calculated^37^ and categorized as trivial (<0.2), small (≥0.2‐0.6), moderate (≥0.6‐1.2), large (≥1.2‐2.0), and very large (≥2.0).^38^

One‐dimensional SPM was used to compare the right arm upper limb kinematics of competitive and recreational handcyclists.^39^ An SPM two‐tailed independent *t* test was used to compare upper limb kinematics at training, competition, and sprint intensities. SPM analysis involves a four‐step process.^40^ Firstly, the scalar test statistic (SPM{t}) was calculated at each data point in the normalized time series. The temporal smoothness of the SPM{t} was then estimated, based on the average temporal gradient. The critical threshold of the SPM{t} was then calculated. SPM uses random field theory correction to ensure that only ≥5% of the SPM{t} data points would reach this significance threshold (α = 0.05) simply by chance had the SPM{t} trajectory resulted from an equally smooth random process.^41^ Finally, the probability (*P*) value of each suprathreshold regions, when SPM{t} exceeds the critical threshold, was calculated. The *P*‐value represents the probability that the observed suprathreshold cluster could have resulted from an equally smooth random process. Detailed examples, theoretical background, and interpretations of SPM statistics are outlined in more detail elsewhere.^39^, ^40^, ^42^ All SPM analyses were conducted using the open‐source spm1d code (v.M0.1, http://www.spm1d.org) in Matlab (R2018a, The Mathworks Inc, Natick, MA).

## 3 RESULTS

Competitive handcyclists flexed their thorax (~5°, *P* < 0.05), extended their shoulder (~10°, *P* < 0.01), and posteriorly tilted their scapula (~15°, *P* < 0.05) more than recreational handcyclists. These significant differences occurred between 40% and 75% of the propulsion cycle. Differences in scapular motion occurred only at competition intensity while differences in shoulder and thorax flexion/extension occurred both at training and competition intensity. No differences in kinematics were found when sprinting due to an increase in SD, particularly in recreational handcyclists. The competitive handcyclists exercised at higher powers and tended to cycle with a higher cadence (ES > 0.6) (Table 2).

**Table 2:** Participant power output, power to weight ratio, and cadence at the training, competition, and sprint intensity (values are Mean ± SD)

Parameter	Competitive	Recreational	ES
Training			
Power (W)	128 ± 8*	99 ± 14	2.63
Cadence (rpm)	91 ± 10	84 ± 14	0.65
Competition			
Power (W)	181 ± 12*	145 ± 19	2.31
Cadence (rpm)	100 ± 13	92 ± 13	0.63
Sprint			
Power (W)	377 ± 59	334 ± 18	0.95
Cadence (rpm)	109 ± 13	100 ± 17	0.62

* *P* < 0.05.

**Figure 1:**
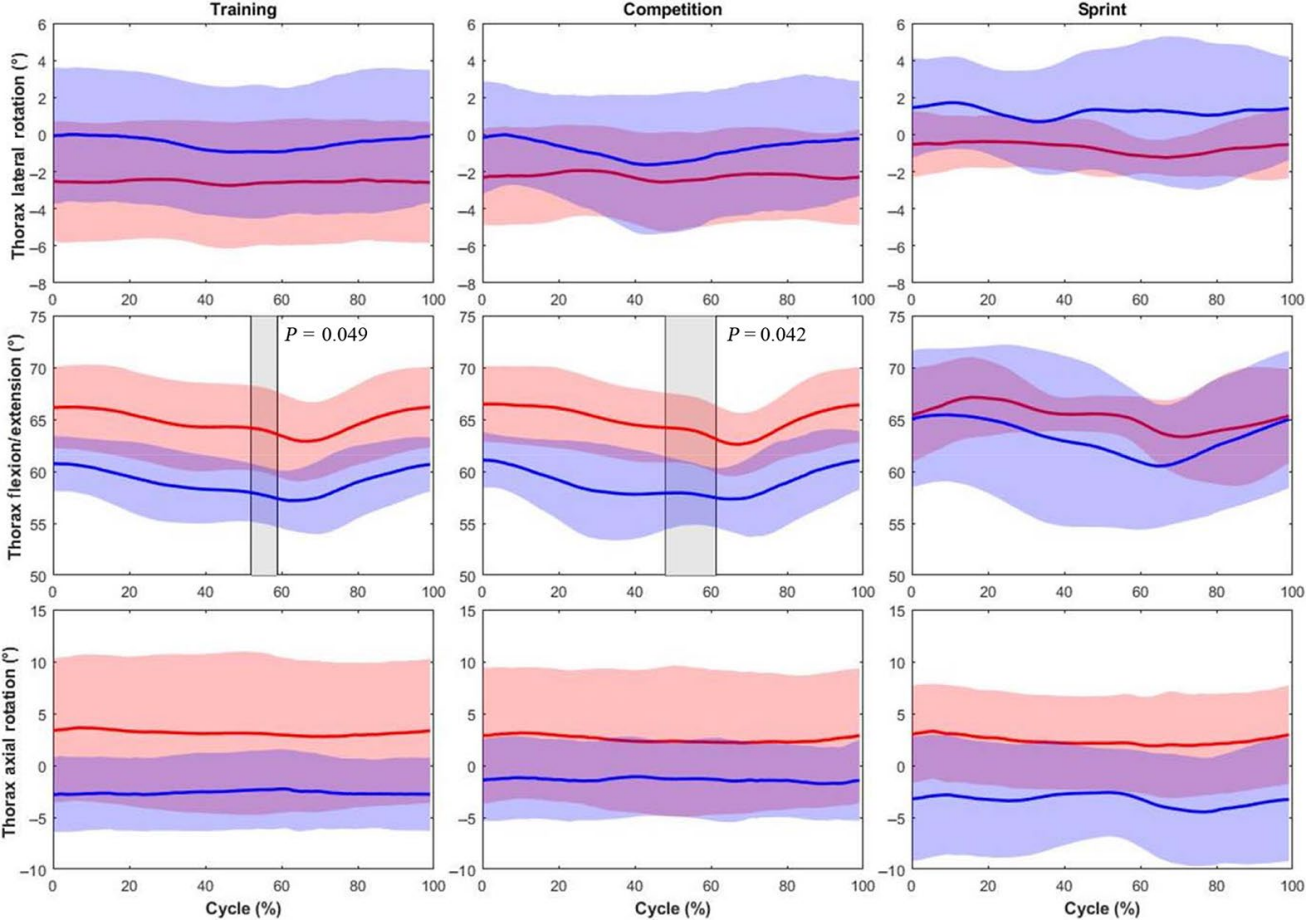
Comparison of the thorax kinematics (group mean kinematic trajectory ± group SD cloud) between competitive (red) and recreational handcyclists (blue) at training, competition and sprint intensities. Shaded regions identify significant differences between groups. P values are provided for each supra‐threshold cluster

Competitive handcyclists tended to have a more flexed thorax (~5°) than the recreational handcyclists throughout the whole propulsion cycle (Figure 1). Significant differences in thorax flexion were identified between 52%‐59% and 48%‐61% (*P* < 0.05) of the propulsion cycle, at training and competition intensities, respectively. No significant differences in axial or lateral thorax rotation were identified. As exercise intensity increased, recreation handcyclists thorax RoM and SD increased in all planes, while the SD reduced and RoM remained constant for competitive handcyclists. However, the 3D RoM for the thorax remained low (<7°) for both groups.

**Figure 2:**
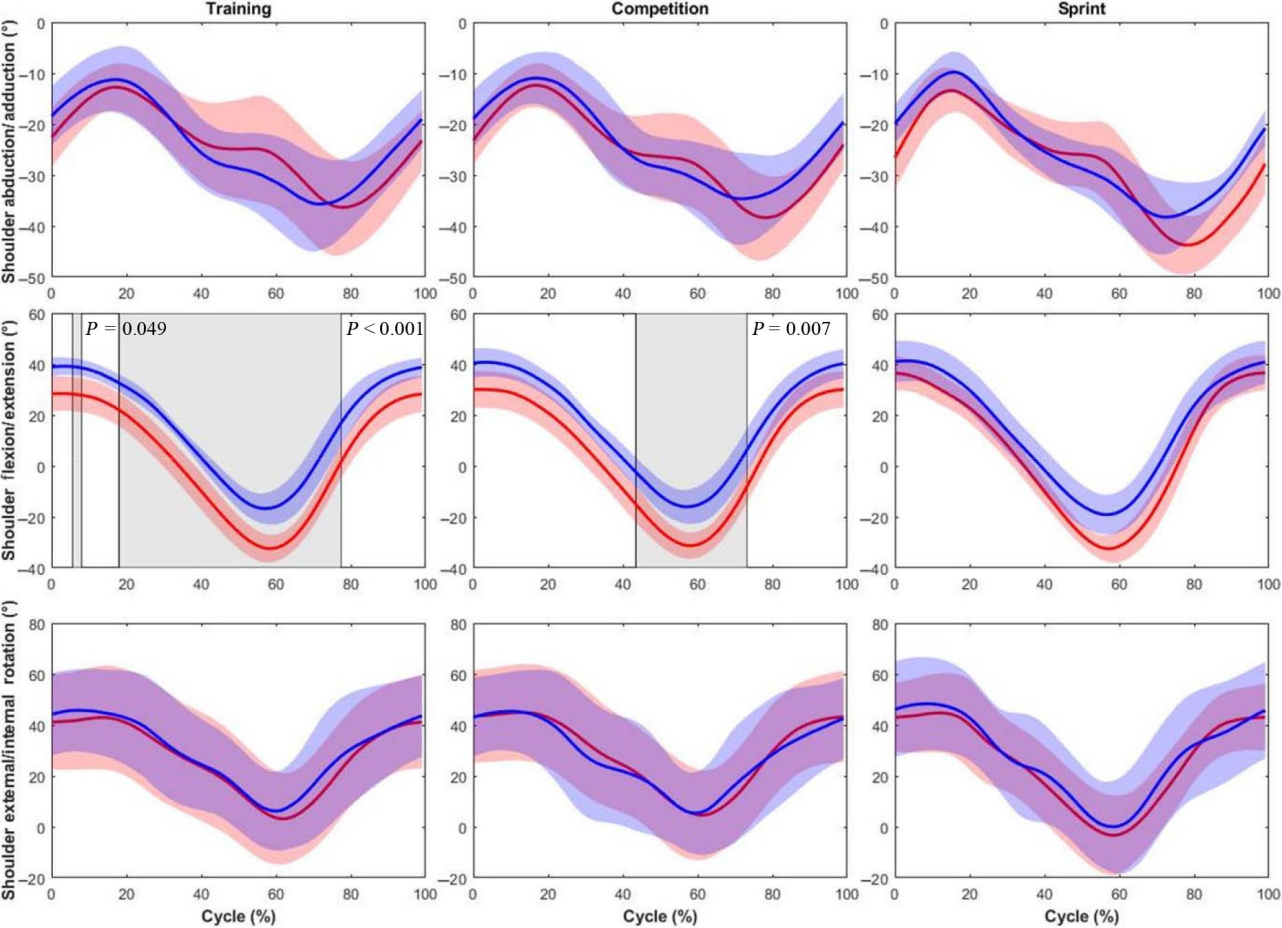
Comparison of the shoulder kinematics (group mean kinematic trajectory ± group SD cloud) between competitive (red) and recreational handcyclists (blue) at training, competition and sprint intensities. Shaded regions identify significant differences between groups. *P* values are provided for each supra‐threshold cluster

**Figure 3:**
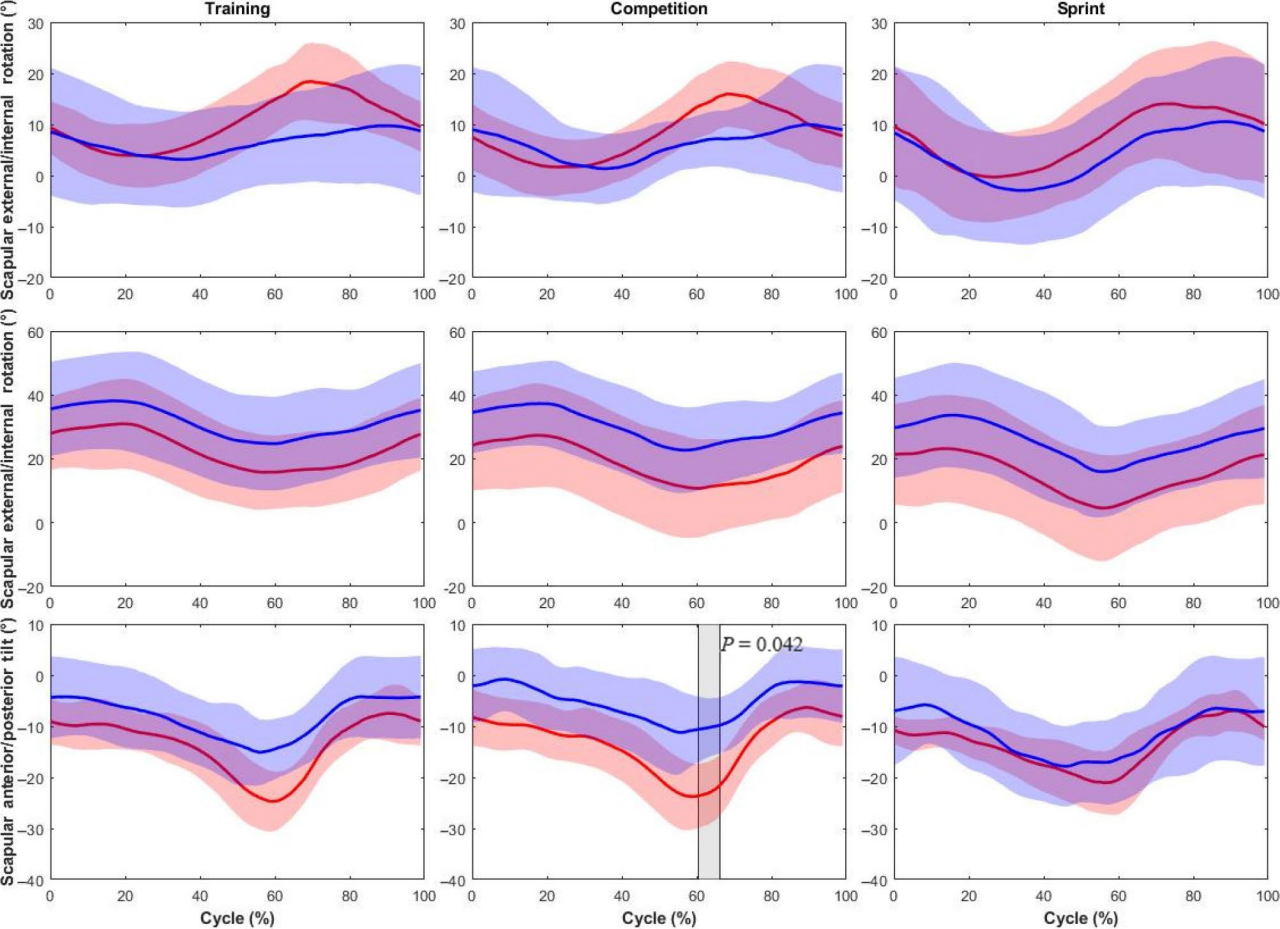
Comparison of the scapular kinematics (group mean kinematic trajectory ± group SD cloud) between competitive (red) and recreational handcyclists (blue) at training, competition and sprint intensities. Shaded regions identify significant differences between groups. *P* values are provided for each supra‐threshold cluster

Greater shoulder extension (~10°) was observed for competitive handcyclists (Figure 2) at training intensity, between 5%‐8% (*P* < 0.05) and 18%‐77% (*P* < 0.001) of the cycle and at competition intensity, between 43% and 73% (*P* < 0.01). Scapular posterior tilt was significantly greater (~15°) between 60% and 66% of the cycle in competitive handcyclists, but only at competition intensity (Figure 3). No other differences in shoulder or scapular motion were revealed.

**Figure 4:**
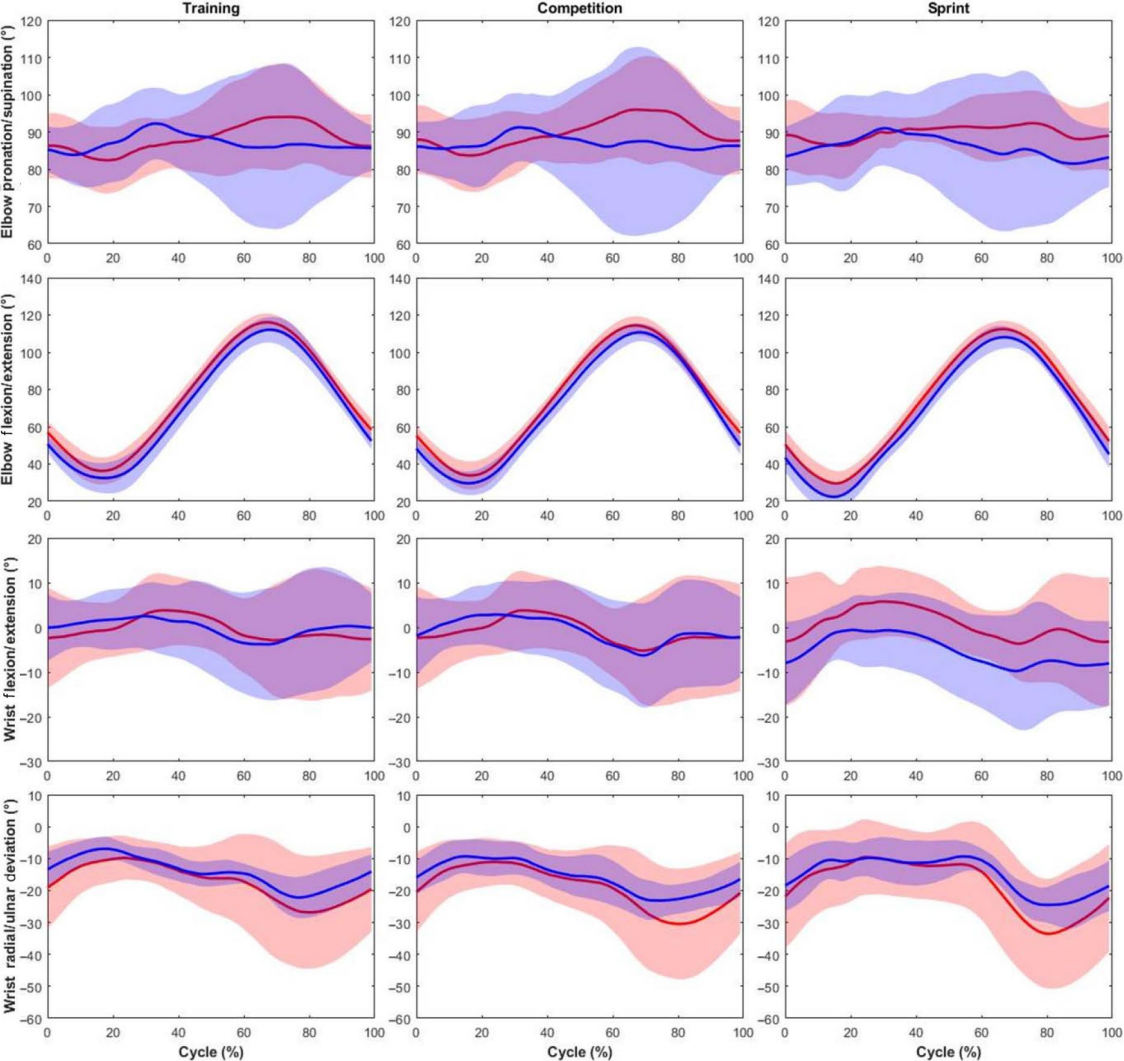
Comparison of the elbow and wrist kinematics (group mean kinematic trajectory ± group SD cloud) between competitive (red) and recreational handcyclists (blue) at training, competition and sprint intensities

No significant differences in elbow or wrist kinematics (Figure 4) were observed across all intensities. Elbow pronation/supination, wrist flexion/extension, and radial/ulnar deviation demonstrated large inter‐individual variability, as evidenced by large SDs, which was particularly noticeable from 50% to 100% of the cycle.

No significant difference in handbike configuration or the handbike‐user interface was identified between groups, also potentially due to the relatively large SDs observed in both groups (Table 3). Competitive handcyclists tended to configure their handbikes with a ~4% greater crank fore‐aft position (ES = 1.04), a lower arm length relative to crank length (ES = 0.90), and a lower arm length relative to crank fore‐aft position (ES = 1.03). Competitive handcyclists had significantly longer arms (*P* < 0.05, ES = 1.25) than the recreational handcyclists.

**Table 3:** Participant anthropometry, handbike configuration, and the configuration of the handbike‐user interface (values are Mean ± SD)

Parameter	Competitive	Recreational	ES
Anthropometry			
AL (cm)	69.4 ± 2.7*	65.9 ± 2.9	1.25
SH (cm)	28.6 ± 2.5	30.2 ± 3.9	0.50
SW (cm)	40.0 ± 1.8	39.2 ± 1.3	0.51
Handbike configuration			
CFAP (cm)	66.0 ± 2.4	63.5 ± 2.4	1.04
CH (cm)	45.9 ± 1.0	46.5 ± 1.6	0.44
CL (cm)	17.2 ± 0.2	17.0 ± 0.5	0.44
CW (cm)	45.5 ± 2.7	44.2 ± 2.0	0.56
Mass (kg)	13.9 ± 0.6	14.4 ± 1.9	0.40
Handbike‐user interface			
SH vs CH (%)	62.2 ± 4.5	64.9 ± 7.9	0.43
AL vs CFAP (%)	95.1 ± 0.9	96.3 ± 1.4	1.03
AL vs CL (%)	24.8 ± 1.1	25.8 ± 1.3	0.90
SW vs CW (%)	88.1 ± 6.1	89.8 ± 3.6	0.33

AL, arm length; CFAP, crank fore‐aft position; CH, crank height; CL, crank length; CW, crank width; SH, shoulder height; SW, shoulder width.

* *P* < 0.05.

## 4 DISCUSSION

The present research provided an opportunity, for the first time, to comprehensively measure handcycling technique and handbike configurations of recumbent handcyclists in sport‐specific conditions and to differentiate between handcyclists from different performance levels (competitive and recreational). The main findings were that competitive handcyclists propelled their handbikes with different propulsion strategies compared to those at a recreational level, despite no differences in handbike configurations. Competitive handcyclists extended their shoulders (~5°) and flexed their thorax (~10°) to a significantly greater extent at training and competition intensities while an increase in scapular posterior tilt (~15°, *P* < 0.05) only occurred at competition intensity. No kinematic differences were observed during sprinting.

Since handcycling is a closed‐chain motion, the configuration of the handbike is likely to affect the technique of the handcyclists.^5^, ^13^, ^19^ It has been suggested that factors such as participant classification and backrest shape affected thorax flexion while the position of the crank axis, crank length, crank width, and the position of the athlete affected posterior scapular tilt and shoulder flexion/extension.^5^, ^13^, ^19^ It is possible that a combination of subtle differences in handbike configuration caused the observed differences in technique between groups. For example, competitive handcyclists had a 2.7% reduction in their shoulder height relative to crank height, potentially affecting shoulder extension, as previously suggested.^13^ Between recreational and competitive groups, participant's classification, handbike configurations, and backrest shapes were comparable, therefore, the observed technical differences may be attributed to the greater skill level the competitive participants.^36^ The greater skill of the handcyclists in the competitive group potentially facilitated their greater power output. Further research is required to determine whether the causes of the observed technical differences are due to differences in handbike configuration, anthropometry or due to differences in technique. Future studies could employ electromyography or cycle kinetics to explore handcycling biomechanics further or determine the differences in technique that occur due to performance level.

Through the use of SPM, the timing of the technical differences in thorax, shoulder, and scapular kinematics were found to occur between ~40%‐75% of the propulsion cycle. This phase of the cycle coincides with the application of high force, termed the pull phase,^34^, ^43^ as the elbow flexes and the shoulder transitions from flexion to extension. The pull phase was one of two distinct phases during the crank cycle where force was applied in handcycling.^43^ The results of the current study suggest that competitive handcyclists employ a different propulsion strategy when compared to recreational users during the pull phase. Potentially, the technical differences observed in the thorax, shoulder, and scapula facilitates force generation during the pull phase (40%‐75% of the cycle) contributing to the increased power output of the competitive handcyclists. Again, further research, examining handcycling kinetics and kinematics, is required to test this hypothesis. However, it was evident that the pull phase is crucial in handcycling and the findings of the current study indicate that coaches and athletes should focus on this phase to optimize technique.

The greatest inter‐individual differences, identified by the SD ranging by >30°, were observed in wrist kinematics and elbow pronation/supination. Similar variability has been reported in the literature, regarding wrist radial/ulnar deviation^13^ and wrist flexion/extension.^5^ Qualitatively, the SDs for the wrist and elbow pronation/supination appeared to be greatest at 50%‐100% of the cycle, as the handgrips pass the thorax and shoulders. During this phase, the shoulder abducts and the elbow transitions from flexion to extension which allows for a greater variation in technique for the forearm and hand. In comparison, between 0% and 50% of the cycle, the forearm and hand are more constrained as the elbows are extended and the shoulders adducted. Therefore, during 50%‐100% of the cycle, a handcyclist has a greater degree of freedom over wrist kinematics and elbow pronation/supination. Also, differences in handgrip size, shape, or angle, which were not measured in the current study, could contribute to this variation. Although there is a high degree of inter‐individual variability in wrist kinematics within the current sample, this variability potentially identifies a variable in handcycling technique that can be coached or altered through training, potentially leading to performance gains or reduced injury risk.

The current study identified that no kinematic differences were observed during sprinting. Interestingly, as exercise intensity increased, recreational handcyclists were less able to maintain a consistent technique. This was evidenced by an increase in RoM and SDs when compared to the competitive handcyclists. Maintaining a stable thorax position was identified to be a critical component of the handbike‐user interface and was perceived to have a substantial impact on performance.^44^ Although sagittal, frontal and transverse plane RoM of the thorax was low (<7°), which was comparable to the 5°‐10° previously reported,^18^ a reduction in thorax RoM could indicate an improvement in technique or handbike configuration. Measuring 3D thorax kinematics could be a useful quantitative tool to measure the stability of a handcyclist in their handbike, which could be beneficial to athletes and coaches when configuring or altering a recumbent handbike.

This study quantified recumbent handbike configuration and athlete anthropometry for the first time and found that competitive handcyclists had significantly longer arms (~3.5 cm) than recreational handcyclists. The greater arm length, of the competitive handcyclists, could contribute the greater power outputs achieved by the competitive handcyclists by increasing the leverage of the arm. No significant differences in handbike configuration or handbike‐athlete interface existed, although this may have been masked by the large inter‐individual differences in handbike configuration. Crank fore‐aft position relative to arm length, crank width relative to shoulder width, and crank height relative to shoulder height varied by 4%, 14%, and 23%, respectively. This variability is potentially due to differences in the physical impairment or that an optimal handbike configuration is yet to be identified as, due to a lack of quantitative data, thus recumbent handcyclists appear to be configuring their handbikes based on trial and error.^14^, ^43^ The authors recommend that future studies physically measure the configuration of the handbike with a tape measure rather than through the analysis of upper limb kinematics as, in the current study, handgrip angle could not be determined and handgrip width was overestimated by ~4 cm (width of hand + marker radius). Using a tape measure would enable consistency with how coaches, support staff and athletes would measure these parameters in the field, hence increasing the transferability of the findings to the “real world.”

The current study was the first study that employed SPM analysis in handcycling, allowing the whole kinematic trajectories to be considered during the statistical analysis. The observed significant differences in thorax flexion and posterior scapular tilt did not coincide with the joint minima or maxima. If independent *t* tests had been used to compare the joint minima, maxima, and RoM, these significant differences might not have been detected. Additionally, if independent *t* tests had been used in the current study, over 100 *t* tests would have been conducted. As SPM considers the entire time series data, the total number of comparisons required to analyze the time series data is substantially reduced.^45^ Furthermore, as SPM considers the entire time series the spatiotemporal context of the biomechanical data is also considered in a theoretically robust manner.^40^, ^42^ Although SPM analysis is robust, few studies have adopted this approach. The results of this study could further emphasize the strengths of SPM in comparison to summary metrics that have been used previously in the handcycling literature.

While this study provided novel data and insights of H3/H4 handcyclists, the sample size was small. However, in the UK the handcycling population is relatively small with the H1 and H2 classes particularly under‐represented. The results of this study are therefore unlikely to be transferable to athletes in the H1 and H2 classes who due to the nature of the impairment, reduced hand and triceps function, could employ a substantially different technique or handbike configuration.

## 5 PERSPECTIVES

This study provided the first comprehensive assessment of recumbent handcycling technique in a valid sporting context. Competitive handcyclists were observed to employ a different propulsion strategy than recumbent handcyclists at training and competition intensities but not during sprinting. Competitive handcyclists extended their shoulders, flexed their thorax and posteriorly tilted their scapular to a greater extent than recreational handcyclists during the pull phase of the cycle.

Athletes and their coaches should technically focus on shoulder extension and thorax flexion during the pulling phase of the propulsion cycle and, due to the inter‐individual variability, wrist, and hand kinematics. These technical components present an opportunity for athletes, their coaches or other support staff to improve performance or reduce the injury risk, through developing novel propulsion strategies or training methods. The insights gained from this study will be of assistance to handbike manufacturers, recumbent handcyclists, their coaches, and physiotherapists.

## References

[1] van der Woude L , Horstman A , Faas P , Mechielsen S , Bafghi HA , de Koning JJ . Power output and metabolic cost of synchronous and asynchronous submaximal and peak level hand cycling on a motor driven treadmill in able‐bodied male subjects. Med Eng Phys. 2008;30:574‐580. 17709272 10.1016/j.medengphy.2007.06.006

[2] Paralympic Games Rio 2016 . Daily competition schedule ‐ Cycling road. Paralympics.org 2016.

[3] Union Cycliste Internationale . Union cycliste internationale cycling regulations ‐ part 16 para‐cycling. 2018;843‐81.

[4] Krämer C , Hilker L , Böhm H . Influence of crank length and crank width on maximal hand cycling power and cadence. Eur J Appl Physiol. 2009;106:749‐757. 19434421 10.1007/s00421-009-1062-1

[5] Litzenberger S , Mally F , Sabo A . Biomechanics of elite recumbent handcycling : a case study. Sport Eng. 2016;19:843‐853.

[6] Abel T , Schneider S , Platen P , Strüder HK . Performance diagnostics in handbiking during competition. Spinal Cord. 2006;44:211‐216. 16172621 10.1038/sj.sc.3101845

[7] Zeller S , Abel T , Strueder HK . Monitoring training load in handcycling: a case study. J Strength Cond Res. 2017;31:3094‐3100. 29068864 10.1519/JSC.0000000000001786

[8] Fischer G , Figueiredo P , Ardigò LP . Physiological performance determinants of a 22 km handbiking time trial of a 22‐km handbiking time trial. Int J Physiol Perform. 2015;10:965‐971. 10.1123/ijspp.2014-0429 25756541

[9] de Groot S , Postma K , van Vliet L , Timmermans R , Valent L . Mountain time trial in handcycling : exercise intensity and predictors of race time in people with spinal cord injury. Spinal Cord. 2014;52:455‐461. 24777165 10.1038/sc.2014.58

[10] Too D . Biomechanics of cycling and factors affecting performance. Sport Med. 1990;10:286‐302. 10.2165/00007256-199010050-00002 2263797

[11] Quittmann OJ , Meskemper J , Abel T , Albracht K , Strüder H . Changes in the kinematic and kinetic profile of handcycling propulsion due to increasing workloads. ISBS Proc Arch. 2017;35:706‐709.

[12] Arnet U , van Drongelen S , Veeger D , van der Woude L . Are the force characteristics of synchronous handcycling affected by speed and the method to impose power? Med Eng Phys. 2012;34:78‐84. 21798789 10.1016/j.medengphy.2011.07.001

[13] Faupin A , Gorce P . The effects of crank adjustments on handbike propulsion: A kinematic model approach. Int J Ind Ergon. 2008;38:577‐583.

[14] Arnet U , van Drongelen S , Schlüssel M , Lay V , van der Woude L , Veeger H . The effect of crank position and backrest inclination on shoulder load and mechanical efficiency during handcycling. Scand J Med Sci Sport. 2014;24:386‐394. 10.1111/j.1600-0838.2012.01524.x 22989023

[15] Lenton JP , Fowler NE , van der Woude L , Goosey‐Tolfrey VL . Wheelchair propulsion: effects of experience and push strategy on efficiency and perceived exertion. Appl Physiol Nutr Metab. 2008;33:870‐879. 18923561 10.1139/H08-072

[16] de Groot S , de Bruin M , Noomen SP , van der Woude L . Mechanical efficiency and propulsion technique after 7 weeks of low‐intensity wheelchair training. Clin Biomech. 2008;23:434‐441. 10.1016/j.clinbiomech.2007.11.001 18077065

[17] Vegter R , De Groot S , Lamoth CJ , Veeger DH , Van Der Woude L . Initial skill acquisition of handrim wheelchair propulsion: A new perspective. IEEE Trans Neural Syst Rehabil Eng. 2014;22:104‐113. 24122567 10.1109/TNSRE.2013.2280301

[18] Faupin A . Kinematic analysis of handbike propulsion in various gear ratios: Implications for joint pain. Clin Biomech (Bristol, Avon). 2006;21:560‐566. 10.1016/j.clinbiomech.2006.01.001 16510220

[19] Faupin A , Gorce P , Watelain E , Meyer C , Thevenon A . A biomechanical analysis of handcycling: a case study. J Appl Biomech. 2010;26:240‐245. 20498497 10.1123/jab.26.2.240

[20] Pataky TC , Robinson MA , Vanrenterghem J . Vector field statistical analysis of kinematic and force trajectories. J Biomech. 2013;46:2394‐2401. 23948374 10.1016/j.jbiomech.2013.07.031

[21] Lovell D , Shields D , Beck B , Cuneo R , McLellan C . The aerobic performance of trained and untrained handcyclists with spinal cord injury. Eur J Appl Physiol. 2012;112:3431‐3437. 22278391 10.1007/s00421-012-2324-x

[22] Beaver WL , Wasserman K , Whipp BJ . Improved detection of lactate threshold during exercise using a log‐log transformation. J Appl Physiol. 1985;59:1936‐1940. 4077801 10.1152/jappl.1985.59.6.1936

[23] Graham‐Paulson T , Perret C , Goosey‐Tolfrey V . Improvements in cycling but not handcycling 10 km time trial performance in habitual caffeine users. Nutrients. 2016;8:843‐853. 10.3390/nu8070393 PMC4963869 27348000

[24] Lovell DI , Mason D , Delphinus E , Mclellan C . A comparison of asynchronous and synchronous arm cranking during the wingate test. Int J Sport Physiol. 2011;6:419‐426. 10.1123/ijspp.6.3.419 21911866

[25] Wu G , Van Der Helm F , Veeger H , et al. ISB recommendation on definitions of joint coordinate systems of various joints for the reporting of human joint motion ‐ Part II: Shoulder, elbow, wrist and hand. J Biomech. 2005;38:981‐992. 15844264 10.1016/j.jbiomech.2004.05.042

[26] Warner MB , Chappell PH , Stokes MJ . Measuring scapular kinematics during arm lowering using the acromion marker cluster. Hum Mov Sci. 2012;31:386‐396. 21875756 10.1016/j.humov.2011.07.004

[27] Shaheen AF , Alexander CM , Bull A . Effects of attachment position and shoulder orientation during calibration on the accuracy of the acromial tracker. J Biomech. 2011;44:1410‐1413. 21306713 10.1016/j.jbiomech.2011.01.013

[28] Warner MB , Whatling G , Worsley PR , et al. Objective classification of scapular kinematics in participants with movement faults of the scapula on clinical assessment. Comput Methods Biomech Biomed Engin. 2015;18:782‐789. 24156508 10.1080/10255842.2013.847093

[29] Warner MB , Chappell PH , Stokes MJ . Measurement of dynamic scapular kinematics using an acromion marker cluster to minimize skin movement artifact. J Vis Exp. 2015;96:843‐14. 10.3791/51717 PMC4354629 25742242

[30] Monnet T , Desailly E , Begon M , Vallée C , Lacouture P . Comparison of the SCoRE and HA methods for locating in vivo the glenohumeral joint centre. J Biomech. 2007;40:3487‐3492. 17631297 10.1016/j.jbiomech.2007.05.030

[31] Taylor WR , Ehrig RM , Duda GN , Schell H , Seebeck P , Heller MO . On the influence of soft tissue coverage in the determination of bone kinematics using skin markers. J Orthop Res. 2005;23:726‐734. 16022983 10.1016/j.orthres.2005.02.006

[32] Ehrig RM , Taylor WR , Duda GN , Heller MO . A survey of formal methods for determining the centre of rotation of ball joints. J Biomech. 2006;39:2798‐2809. 16293257 10.1016/j.jbiomech.2005.10.002

[33] Cappozzo A , Catani F , Della Croce U , Leardini A . Position and orientation in space of bones during movement. Clin Biomech. 1995;10:171‐178. 10.1016/0268-0033(95)91394-t 11415549

[34] Verellen J , Janssens L , Meyer C , Vanlandewijck Y . Development and application of a handbike ergometer to measure the 3D force generation pattern during arm crank propulsion in realistic handcycling conditions. Sport Technol. 2012;5:56‐73.

[35] Jennings A . Sphere Fit (least squared) [software]. Matlab Cent File Exch. 2013.

[36] Bini RR , Dagnese F , Rocha E , Silveira MC , Carpes FP , Mota CB . Three‐dimensional kinematics of competitive and recreational cyclists across different workloads during cycling. Eur J Sport Sci. 2016;16:553‐559. 26783692 10.1080/17461391.2015.1135984

[37] Cohen J . Statistical power analysis for the behavioral sciences. Hillsdale, NJ: Associates., Lawrence Earlbaum; 1988; 567.

[38] Batterham AM , Hopkins WG . Making meaningful inferences about magnitudes. Int J Sports Physiol Perform. 2006;1:50‐57. 19114737

[39] Friston KJ , Ashburner JT , Kiebel SJ , Nichols TE , Penny WD . Statistical parametric mapping: the analysis of functional brain images. Amsterdam: Elsevier; 2007.

[40] De Ridder R , Willems T , Vanrenterghem J , Robinson M , Pataky T , Roosen P . Gait kinematics of subjects with ankle instability using a multisegmented foot model. Med Sci Sports Exerc. 2013;45:2129‐2136. 23657166 10.1249/MSS.0b013e31829991a2

[41] Adler RJ , Taylor JE . Random fields and geometry. New York: Springer‐Verlag; 2007.

[42] Pataky TC . Computer methods in biomechanics and biomedical engineering one‐dimensional statistical parametric mapping in Python. Comp. 2012;15:295‐301. 10.1080/10255842.2010.527837 21756121

[43] Krämer C , Schneider G , Böhm H , Klöpfer‐Krämer I , Senner V . Effect of different handgrip angles on work distribution during hand cycling at submaximal power levels. Ergonomics. 2009;52:1276‐1286. 19626501 10.1080/00140130902971916

[44] Stone B , Mason BS , Bundon A , et al. Elite handcycling: A qualitative analysis of recumbent handbike configuration for optimal sports performance. Ergonomics. 2018:843‐25. 10.1080/00140139.2018.1531149 30281401

[45] Knudson D . Significant and meaningful effects in sports biomechanics research. Sport Biomech. 2009;8:96‐104. 10.1080/14763140802629966 19391497

